# Perceived health and well-being of migrant female street food vendors: a mixed – method study of vendor – consumer perceptions of informal street food economy in Chennai, India

**DOI:** 10.3389/fpubh.2026.1918016

**Published:** 2026-10-07

**Authors:** A. Wincie Wilmah, R. K. Jaishree Karthiga

**Affiliations:** School of Social Sciences and Languages, Vellore Institute of Technology, Chennai Campus, Chennai, India

**Keywords:** female vendors, health, migration, street food, Sustainable Development Goal 3, well-being

## Abstract

**Background:**

The phenomenon of migration has impacted a country’s economy in significant ways. This study aims to understand the impact of migration on the health and well-being of migrant female street vendors through a mixed-method research design.

**Methods:**

The quantitative research focuses on statistical analysis of *n* = 40 responses from the consumers who purchase from the migrant female street food vendors. The qualitative research involves conducting interviews based on the Interpretive Phenomenological research method from four female migrant street food vendors in Urban Chennai, Tamil Nadu, India.

**Results:**

The statistical analysis of the six-item scale focusing on the health and well-being of the vendors yielded highly significant values (*p* < 0.005) and a Cronbach’s Alpha value of (*a* = 0.75). The qualitative analysis resulted in three major themes, which focused on physical exhaustion, illnesses, and communication barriers observed through the vendor responses.

**Conclusion:**

The qualitative and quantitative results were triangulated to provide findings that emphasized the need to understand and create policies that would enhance the overall quality of life of these migrant communities. These findings collectively aim to emphasize that the needs of Sustainable Development Goal 3, of Good Health and Well-being, in relation to the informal work sectors are efficiently met.

## 1 Introduction

The urban informal food sector provides a crucial ground for the migrant and marginalized groups to enhance their survival strategy (1). The informal food sectors dominate the urban landscape by providing food security through regular community activities rather than merely functioning as survival strategies for the migrants (2). The informal street food markets are a representation of urban inhabitation, which challenge the elite food markets through the spatial knowledge acquired by the vendors, which also enables them to navigate the conditions of precarity arising because of operating within a non- neutral commercial framework (3). Though the informal street food economy acts as a support for the migrants and small-scale business owners to navigate hierarchical marginalization and poverty (2), the sector operates with its own hardships for the vendors, such as socio-cultural, economic, and infrastructural hardships (4).

The major implication prevailing in the informal street food economy is the health and well-being of the migrant communities. The migration pattern affects the social, political, economic and physical well-being of the migrants due to the friction between the migrant society and the host environment, particularly seen in the food patterns and culture of the migrants (5). The state of migration leads to visible disparities, especially in informal small-scale economies or street vendor businesses, where the migrant vendors tend to face more difficulties than the local/native vendors (1). The mental and physical well-being of the women internal migrants engaged in informal street-food vending vendors is easily subjected to a state of vulnerability due to the lack of alternate employment opportunities, barriers to access structured organizations due to language and cultural differences, and a significant lack of knowledge regarding the host environment (1). The concerns regarding the socio-economic and cultural vulnerabilities of the migrants are further inflated by the serious concerns of health issues and crises revolving around migrant well-being. The COVID-19 pandemic, for example, resulted in long-term lockdowns, reduced access to street vending by raising health concerns from roadside food businesses, thereby leading to an eventual loss of the source of income for the migrant vendors, and a serious impact on their mental and physical health conditions (1, 6, 7).

The migrant health, in the case of street food vendors, especially female vendors, is often threatened by the hazards of the open public spaces, which impact their lives both directly and indirectly. The infrastructural drawbacks in the street food vending zones are inadequate sanitary measures, lack of social and public health security measures and difficulties in accessing clean public restrooms and other similar issues of concern (4). The hostility in the urban environments influences the vendors to adapt cost-efficient measures such as pushcarts, rickshaws and long-standing work hours, which leads to their prioritization of commercial gain rather than their own safety (3). The lack of a formal food safety assistance for these small-scale migrant vendors poses an impending health hazard for both the vendors and the consumers due to improper education regarding food handling and storage measures (4, 8).

The issues of health and livelihood risks are further influenced by the status of migration and gender. The existing demographics reveal that the informal urban food sector is highly feminized, with a huge population of street food vendors being women (6, 8). The biased gender role expectations placed on women force them to navigate the double-burdens of workplace inequalities and unshared household duties, which raises a serious concern regarding the health conditions of these women (6, 9). The statistical data reveal that the migrants in the domestic sector are often subjected to conditions of poverty and malnutrition more than the native vendors in the sector, which raises a serious concern to address the issue of health and well-being of these migrant women communities (9).

The migrant populations find it difficult to navigate through their dietary practices due to the differences in societal and cultural practices, economic constraints and discrimination (5). There is a crucial gap prevailing in existing literature concerning these migrant female street food vendors due to the structural limitations and westernization of data collection models, which fail to address and capture the lived experiences of the local migrant population (10).

The aim of this study is to address the gap prevailing in existing research by studying the lives of migrant female street food vendors in Chennai, by analyzing the perspectives of both vendors and consumers with relevance to the health and well-being of these migrant communities. The study aims to address this issue in accordance with Sustainable Development Goal 3 by emphasizing that the informal street food vendors are also entitled to ensure good health and well-being. The study focuses on the intersection of the health and well-being of these female migrants by integrating consumer survey responses with a qualitative, in-depth phenomenological study of the lived experiences of migrant female street food vendors in Chennai from inter-state and intra-state migration status. This mixed-method design aims to provide a comprehensive, multi-perspective inquiry into the occupational hazards and socio-cultural barriers faced by these vendors. The research examines physical incapacities, chronic illnesses, language barriers and migration statuses as the primary causes for the vulnerabilities and deteriorating health and well-being of the migrant female street food vendors. The research ultimately aims to persuade policymakers to provide sensitive measures towards enhancing the lives of these migrants by considering the informal street food sectors as a key site of economic enhancement and to prioritize the inclusion of migrant women in policy-making and urban development planning (1, 2, 10).

## 2 Methodology

The study combines qualitative phenomenological interviews with an internally validated quantitative survey to analyze the physiological well-being of the migrants. This convergent mixed-method design evaluates the macro-level perspectives through consumer surveys along with the micro-level perspectives of migrant narratives to provide a comprehensive analysis of the phenomena under study. Integrating consumer perspectives into an occupational health study on informal migrant street vendors serves a critical methodological and triangulation purpose.

In the informal street food economy, migrant female vendors operate in public spaces under high economic and social precarity. The migrant female vendors might not report their struggles or sufferings accurately due to the fear of social representations, loss of regular customers or other legal issues. Therefore, these struggles could be observed by the consumers who interact with the vendors every day and therefore become passive observers of their health and well-being. The consumers are able to observe physical signs of exhaustion of the vendors, such as frequent rubbing of the neck/limbs, respiratory illnesses arising due to excessive heat and linguistic barriers. The triangulation of vendor narratives (*N* = 4) with the consumer perceptions (*N* = 100) offers a comprehensive lens to examine the health and well-being of the vendors while simultaneously minimizing self-reporting bias and therefore significantly contributing towards understanding the social conditions of the informal urban food economy.

### 2.1 Quantitative research design

The urban residents in Chennai are often identified to be regular consumers of street food, especially from shops belonging to the informal urban food sector. Consequently, they serve as daily, third-party observers who can compare and contrast the groups across key indicators, such as language-induced customer friction, vulnerability to administrative harassment, and overall work-related stress. The native consumers act as a bridge to establish a connection between the non-native vendors and the native vendors as they consume from both the vendors, thereby rejecting the need for the study to specifically include the native vendor samples. To operationalize the comparative assessment between native and migrant vendors, the quantitative scale incorporates Question 6 (“Consumer opinion on increased stress levels among migrant vendors compared to native vendors”). Since the primary participant pool is composed of migrant women, consumers act as a comparative proxy. This allows the study to capture comparative socio-economic precarity without requiring a separate native vendor sample.

The quantitative scale used to measure the consumer responses is a key element of this study. Because the survey respondents are consumers rather than the vendors themselves, this component does not capture clinical, pathological, or self-reported medical data (such as formal diagnoses of chronic respiratory diseases or musculoskeletal disorders). Instead, the quantitative tool is strictly operationalized as a survey of consumer perceptions and externally observable markers of physical and operational strain. Consumers serve as proxy observers of overt, outward somatic reflexes (such as heavy breathing, physical slumping, coughing fits, and communication-related frustration) during active street transactions. Consequently, all quantitative findings are presented as perceived or observed indicators of vulnerability rather than objective clinical evidence, and we explicitly avoid drawing causal assertions regarding the direct impact of migration on biological health.

#### 2.1.1 Survey questionnaire design, pilot testing, and optimization

The quantitative survey was designed to capture public consumer perceptions of the physical, socio-linguistic, and operational vulnerabilities of migrant female street food vendors in Chennai. The Google Forms questionnaire was administered exclusively in English, as it was distributed online to urban consumers across Chennai via social media platforms.

The initial pilot questionnaire designed for the study consisted of 18 questions measuring broad physical, structural, social, and cultural barriers. This pilot instrument was convenience-sampled among 10 consumers, yielding an initial Cronbach’s Alpha of 0.606. A qualitative item-by-item analysis revealed that 12 of the pilot items relied heavily on parameters that consumers could not directly or reliably observe during brief retail transactions (such as “The vendor sleeps at least 7 h a day,” “The vendor has active health insurance,” or “The vendor experiences extreme business debt”). Because consumers could not answer these items accurately, they introduced high measurement noise. Therefore, these 12 items were removed, and the scale was optimized into a refined 6-item set focusing exclusively on observable, external indicators of somatic and operational precarity.

These 6 finalized items were evaluated on a 5-point Likert scale (where 1 = Strongly Disagree, 2 = Disagree, 3 = Neutral, 4 = Agree, and 5 = Strongly Agree). The exact English item wordings and their scoring directions are detailed below:

1. The opinion of the consumers regarding the perceived physical incapability of the vendors while handling the physical demands of street food vending. Likert scale Score: 1 to 5 (Higher score indicates higher perceived physical incapability/vulnerability).
2. The observation of the consumer regarding the prevalence of visible signs of physical exhaustion (e.g., slumping, heavy breathing) in the vendor. Likert scale Score: 1 to 5 (Higher score indicates higher observed somatic exhaustion).
3. Symptoms of chronic pain (e.g., frequent rubbing of neck, legs, and hands) observed in the vendor. Scoring: 1 to 5 (Higher score indicates higher observed musculoskeletal strain).
4. The consumer’s observation on the visible signs of the vendors regarding symptoms of respiratory diseases (e.g., persistent coughing, wheezing, sneezing, or labored breathing near the cooking stove). Likert scale Score: 1 to 5 (Higher score indicates higher observed respiratory distress).
5. The struggles of vendors as observed by the consumers regarding Linguistic or communication barriers which leads to visible frustration in the vendor. Likert scale Score: 1 to 5 (Higher score indicates higher observed socio-linguistic precarity).
6. Increased operational and survival hardships faced by migrant vendors compared to local (native) vendors. Scoring: 1 to 5 (Higher score indicates higher perceived comparative hardship).

Methodological Justification of the Construct: Because these six items evaluate distinct aspects of a vendor’s environment: somatic strain, physiological distress, linguistic friction, and comparative hardship they do not form a strict, unidimensional psychological construct. Therefore, treating them as a single, additive scale would be conceptually fragile. Instead, we treat these items as independent exploratory markers representing different dimensions of “Perceived Socio-Environmental and Physical Precarity” under SDG 3. To maintain methodological rigor, we do not aggregate the items into a single composite score. Instead, we analyze each item separately using non-parametric One-Sample Wilcoxon Signed-Rank tests, and we examine their co-occurrence through targeted bivariate Spearman rank correlation pairs (e.g., Q1–Q2 for exhaustion; Q3–Q4 for environmental illness; Q5–Q6 for linguistic-survival barriers). While the overall 6-item scale reliability of alpha = 0.75 is reported, it is presented purely as a descriptive indicator of the items’ shared focus on vendor vulnerability rather than an assumption of one-dimensionality.

### 2.2 Survey responses and sample size

#### 2.2.1 Survey responses and sample selection flow

The Google Forms survey received *N* = 100 initial responses from the public. However, to ensure methodological rigor, we employed a strict three-stage screening process to exclude cases that did not adhere to the defined eligibility criteria. Cases were excluded not based on statistical performance but based on predetermined inclusion criteria. The exact attrition of the survey sample is described below:

1. Step 1: Screening for missing or incomplete data (n = 15 excluded).

We excluded cases with missing, blank or incomplete questionnaire items or data, particularly on the core Likert item scales.

1. Step 2: Screening for response unreliability and inattentiveness (*n* = 20 excluded).

To ensure the quality of our data, we have excluded cases containing “unreliable” responses. We operationalized “unreliable” responses using the following criteria: (i) Contradictory responses (selecting “Strongly Agree” for Q1, Perceived Physical Incapability, while selecting “Strongly Disagree “for Q2, Physical Exhaustion and self-contradictory, random responses).

1. Step 3: Screening for violation of inclusion criteria (*n* = 25 excluded).

We excluded cases that did not meet our target population of interest. To be included in our analysis, the consumer had to: (a) reside in Chennai, and (b) purchase food directly from a migrant female street food vendor in Chennai within the last 6 months. Consumers who only purchased from native vendors, male vendors, or outside Chennai were excluded.

Following this screening process, we had a final analytical sample of n = 40 highly reliable and eligible responses. The internal consistency of the final 6-item scale was then calculated on this cleaned dataset, resulting in a Cronbach’s Alpha of 0.75. Notably, the exclusion of cases was performed to ensure the quality of data and the eligibility of the sample in terms of demographics prior to conducting any statistical analyses; no cases were excluded to artificially increase the reliability coefficient.

The sufficient size of our n = 40 was validated using a post-hoc statistical power analysis (alpha = 0.05). Given the lowest observed significant correlation 0.637, our post-hoc power analysis produced an extraordinary value of 0.99999916, confirming that our study is highly powered and completely protected against Type II statistical errors.

### 2.3 Quantitative analysis

We had used a manual analysis through the Microsoft Excel spreadsheet to analyze the collected quantitative data. The analysis was performed in the following stages.

#### 2.3.1 Inferential statistical tests

The collected quantitative data were then analyzed by performing the following tests:

Spearman’s Rank Correlation test was performed to find the correlation that exists between two factors in relevance to the vendors’ health and well-being. The tests were performed between the following criteria:

1. Perceived Physical Incapability of handling the demands and visible signs of exhaustion.
2. Association between chronic pain and respiratory illness of the vendor.
3. Impact of language barriers and the hardships encountered by the migrant vendors as compared to the native vendors.

1. A one-sample t-test was performed on Question 6 to assess the consumer’s perspective on the difficulties faced by migrant vendors as compared to the native vendors. Since the variable was measured using a 5-point Likert scale, the sample mean was tested against a hypothesized neutral value of 3.0 (representing “no perceived difference in hardship levels between native and migrant vendors”). A sample mean significantly higher than 3.0 indicates a statistically validated consensus that migrant vendors face a significantly higher degree of operational and survival precarity than native vendors.

### 2.4 Qualitative research methodology

#### 2.4.1 Epistemological framework and IPA commitment

The research uses a qualitative idiographic Interpretative Phenomenological Analysis (IPA) that will be based on the theoretical framework of Smith, Flowers, and Larkin’s (12). The choice of IPA over standard thematic analysis is justified by the former’s double-hermeneutic nature that involves “the researcher making sense of the participant making sense of their world” (Smith, Flowers, & Larkin, 2022, p. 8). Although both qualitative methodologies are aimed at discovering themes and patterns in the collected data, IPA stresses the need to study every participant’s story using an idiographic approach before shifting to the nomothetic one.

#### 2.4.2 Participant recruitment and sample profile

In line with IPA’s recommendation of a small sampling strategy (typically 3 to 6 participants) and the need for a homogenous sample, we recruited four migrant female street food vendors (*N* = 4) in Urban Chennai, Tamil Nadu, India. Homogeneity was established using four strict inclusion criteria: (i) self-identification as a woman; (ii) acting as a primary owner or operator of an informal roadside food stall or pushcart; (iii) residing and working in Chennai; and (iv) having an active migration background (inter-state or intra-state). Recruitment was conducted using on-site street outreach in commercial vending hubs in Chennai. In accordance with IPA’s idiographic commitment, the four case studies represent diverse migration backgrounds:

- Participant 1 (P1): A 34-year-old inter-state migrant from Assam (operating a street stall selling North-Eastern/fast food for 6 years; non-Tamil speaker).
- Participant 2 (P2): 38 years old interstate migrant from West Bengal (Snack/beverage cart operator for 8 years; limited Tamil).
- Participant 3 (P3) 52 year old inter-state migrant from Kerala (running traditional breakfast/meal stall for 15 years; fluent in conversational Tamil due to linguistic proximity with Malayalam).
- Participant 4 (P4): 40 year old intra-state migrant from Chidambaram, Rural Tamil Nadu (operating a tiffin pushcart for 15 years; native Tamil speaker).

#### 2.4.3 Interview setting, duration, and data collection protocol

The primary investigator conducted in-person, semi-structured interviews. To avoid inconveniencing the vendors businesses and enhance their psychological wellbeing, the interviews were held directly at their vending stalls during non-business hours between 3:00 p.m. and 5:00 p.m. Each interview session lasted between 35 min and 50 min. The semi-structured interview guide comprised of open-ended, non-directive questions pertaining to four thematic areas namely: (a) Migration history and transition into city; (b) Working routines and physical labor; (c) Physical health manifestations, inhalation of stove-smoke, and chronic body pains; (d) Language barriers, interpersonal communication, and personal safety. The interviews were audio-recorded using a high-fidelity digital recorder with supplementary field notes capturing paralinguistic information such as long pauses, sighs, and momentary hesitations.

#### 2.4.4 Transcription, translation, and rigor

The interviews were conducted in Tamil (the lingua franca of Chennai). The primary researcher began by transcribing the Tamil interviews verbatim in order to retain local idioms, linguistic nuances, and colloquialisms. The transcripts were then translated to English for the purposes of coding. In order to affirm the accuracy of the cross-translation and ensure conceptual equivalence, an independent bilingual expert in social sciences performed the reverse procedure (back-translation) of the interview transcripts from English to Tamil. Any discrepancies were ironed out in consensus. Qualitative rigor and trustworthiness were enhanced through:

- Member-Checking: Excerpts of the translated interview transcripts and key verbatim quotes were shared and discussed with the four vendors during a subsequent visit to their vending site in an effort to confirm that the researcher has accurately understood their lived experiences.
- Audit Trail: An elaborate audit-trail was generated through systematic documentation of all analytic procedures, including verbatim transcripts, reflexivity journals, initial codebooks, and emerging thematic clusters.

#### 2.4.5 Researcher reflexivity

The researcher is the primary instrument in qualitative phenomenological research. The primary investigator is an educated bilingual female academic who resides in Chennai. This raises issues of power relations between an academic researcher and vulnerable migrant workers. Consequently, the researcher kept a reflexivity journal throughout the study in order to track and manage any preconceptions or subjective ideologically-informed interpretations that might interfere with the authenticity of the participants’ voices. Reflexive bracketing was employed by deliberately stating one’s pre-research understandings about the migrant crisis in order to consciously separate such assumptions from the meanings that the participants intended to convey.

#### 2.4.6 Step-by-step idiographic IPA analytical process

The analysis followed the six stages of the Idiographic Interpretative Phenomenological Analysis framework as proposed by Smith, Flowers and Larkin (12):

1. Stage 1: Reading and re-reading the raw interview files and transcript of participant to familiarize oneself with the world view of the interviewee.
2. Step 2: The researcher coded the interview transcripts line by line using three different types of codes: Descriptive codes (pertaining to the overt content and salient happenings), Linguistic codes (pertaining to language use, pauses and laughter) and Conceptual codes (involved in-depth reflections pertaining to the psychological implications of the phenomenon).
3. Stage 3: Condensing of initial inductive codes to form emerging psychological themes.
4. Stage 4: Finding relations between emerging themes by ordering them in a hierarchy of conceptual categories through spatial arrangement, abstraction and polarization.
5. Stage 5: Moving on to the next case (idiographic commitment). The researcher detached from the previous interview, in order to avoid carryover effects and to concentrate on the upcoming interview transcript.
6. Stage 6: Looking for across-case patterns (cross-case hermeneutics). The researcher performed a process of cross-case synthesis to arrive at superordinate themes while simultaneously acknowledging and retaining idiographic variations (e.g., linguistic differences between inter-state and intra-state migrant respondents.)

#### 2.4.7 Ethical approval, informed consent and the privacy of participants

Institutional Ethical Approval: The present study has been approved by the Institutional Ethical Committee For Studies on Human Subjects (IECH) of Vellore Institute of Technology (VIT), Chennai Campus. Approval Reference Number: VIT/IECH/2025/85. Date of Approval: 7 th October 2025.

Distinctive consent procedures:

Online Consumer Survey: The online informed consent procedure was embedded in the online survey (Google Forms). Before answering the 6 Likert scale items, the participants could view an information sheet with information on the scope of the research, voluntary participation, and the confidentiality of the data. The participants were required to explicitly mark the option I agree to participate before proceeding to the survey. No Personally Identifiable Information (PII) and Internet Protocol (IP) addresses of the participants were collected.

In-Person recorded interviews: A separate verbal consent procedure was conducted in Tamil before the qualitative interviews. The principal investigator read through the consent form in Tamil informing the potential participant about the details of the study, voluntary participation, and the right to withdraw at any time without penalty. All vendors confirmed that they were consenting adults (18 years of age or older). For the participants with low levels of formal education, their verbal consent was audio-recorded and a thumb impression or signature was obtained on the consent form in the presence of an independent witness.

Participant privacy and anonymity safeguards: As the qualitative study’s participants are four vendors identified by their region/state of origin, a few privacy safeguards have been taken to ensure confidentiality and anonymity, including:

1. In the study, the participant’s names have been replaced by alphanumeric codes (Participant 1 to Participant 4/ P1 to P4).
2. The street stalls and landmark’s exact names and GPS location have been removed from the final output of the study. In addition, the street vendors’ working locations have been mentioned as Urban Chennai. However, their city of origin has been mentioned as Assam, West Bengal, Kerala, and Chidambaram.

## 3 Results

This section presents the findings of this mixed-method study, which integrates the macro-level consumer perception of the Migrant female street food vendors (*n =* 40) with the micro-level Interpretative Phenomenological Analysis of the migrant vendor narratives (*N* = 4).

### 3.1 Quantitative results

The quantitative survey for consumer perceptions (n = 40) was analyzed using both parametric (Pearson’s r) and non-parametric (Spearman’s rho) correlation coefficients simultaneously in order to maximize robustness and ensure transparent cross-validation. For each bivariate correlation, the exact t- statistic was calculated with 38 degrees of freedom (*df* = N−2) using standard formula t = r sqrt ((N−2)/(1−r^2^)), and its exact two-tailed *p*-value.

Table 1 shows a perfect positive monotonic relationship (Spearman’s = 1.000), between consumers’ perception of the Perceived Physical Incapability of the vendors, (Q1) and visible externally observed indicators of physical exhaustion (Q2). This means that there is a very high degree of agreement amongst consumers that the physical nature of the roadside vending is associated with external somatic strain amongst female vendors.

**Table 1:** Correlation between perceived physical incapability (Q1) and observed physical exhaustion (Q2).

Metric	Pearson correlation (*r*)	Spearman correlation (*ρ*)
Coefficient value	1.0000	1.0000
*t*-statistic (*df* = 38)	Undefined (∞)	Undefined (∞)
*p*-value (2-tailed)	<0.0001	<0.0001
Significance level	Highly significant (*p* < 0.001)	Highly significant (*p* < 0.001)

In raw survey data, consumer ratings for Q1 (Perceived Physical Incapability) and Q2 (Physical Exhaustion) are identical across all 40 respondents, yielding a mathematically perfect positive correlation of 1.0000. This reflects an absolute, unanimous consumer consensus that a vendor’s physical incapability to meet roadside vending demands is perfectly correlated with visible somatic exhaustion.

As shown in Table 2, there is a strong statistically significant positive correlation (Spearman’s = 0.7962, *p* < 0.05) between observed chronic pain (Q3) and symptoms of respiratory illness (Q4). The high significance of this association identifies that physical musculoskeletal strain (pain from posturing during prolonged standing) co-occurs with acute respiratory distress (coughing/wheezing from cooking smoke and road emissions) signalling a compounded occupational health burden.

**Table 2:** Correlation between observed pain posturing (Q3) and observed symptoms of respiratory distress (Q4).

Metric	Pearson correlation (*r*)	Spearman correlation (*ρ*)
Coefficient value	0.8194	0.7962
*t*-statistic (*df* = 38)	8.8122	8.1122
*p*-value (2-tailed)	1.01 × 10^−10^	8.12 × 10^−10^
Significance level	Highly significant (*p* < 0.001)	Highly significant (*p* < 0.001)

Table 3 shows a positive and statistically significant correlation (Spearman’s = 0.6373, *p* < 0.005) of a moderate-to-strong degree between observed communication barriers (Q5) and perceived harder survival conditions in comparison with native vendors (Q6).

**Table 3:** Correlation between observed communication barriers (Q5) and perceived survival conditions (Q6).

Metric	Pearson correlation (*r*)	Spearman correlation (*ρ*)
Coefficient value	0.5897	0.6373
*t*-statistic (*df* = 38)	4.5010	5.0977
*p*-value (2-tailed)	6.22 × 10^−5^	9.78 × 10^−6^
Significance level	Highly significant (*p* < 0.001)	Highly significant (*p* < 0.001)

To assess the public perception of the relative levels of hardship faced by migrant female and local native vendors, a parametric One-Sample t-test procedure was conducted on Question 6 against a neutral benchmark value of 3.00 (corresponding to “Neutral/No Perceived Difference” on the 5-point Likert scale: 1 = Strongly Disagree, 3 = Neutral, 5 = Strongly Agree). The findings are summarized in Table 4.

**Table 4:** One-sample t-test for hardships encountered by the migrant vendors (Q6).

Parameter	Value
Sample mean (x-bar)	3.5250
Population baseline (mu_0)	3.0000 (neutral midpoint)
Sample standard deviation (s)	0.8161
Sample size (N)	40
Standard error of the mean (SE)	0.1290
Degrees of freedom (*df*)	39
*t*-statistic	4.0686
95% Confidence interval for the mean	[3.2640, 3.7860]
Exact p-value (2-tailed)	0.000223 (highly significant)
Effect size (Cohen’s d)	0.6433 (medium-to-large effect)

The one-sample *t*-test results in Table 4 demonstrate a highly significant *t*-statistic value (*t* = 4.07, *df* = 39, *p* < 0.001). This reveals that the sample mean (x-bar = 3.5250) differs significantly from the neutral benchmark of 3.0. The 95% confidence interval [3.2640, 3.7860] excludes the neutral value, and the Cohen’s d effect size of 0.6433 indicates a large effect. This finding confirms that consumers strongly agree (mean > 3.5) that migrant female street food vendors in Chennai face substantially more precarious living and working conditions than local native vendors.

### 3.2 Qualitative results

The qualitative analysis of the migrant female street food vendors’ interview transcripts was manually analyzed and coded thematically based on common ideas and experiences encountered by the vendors. The following Table 5 provides the tabulation of the select responses provided by the vendors.

**Table 5:** Tabulation of migrant female vendor responses.

Superordinate theme	Sub-themes/core experience	Primary participant evidence	Idiographic divergence/case nuance
Theme 1 somatic strain & exhaustive work cycles	Unshared double-burden; sleep deprivation; constant standing	P4: 4:30 a.m. to 12:00 a.m. cycle; pushcart labor. P3: Age-related physical collapse after 15 years.	P4 experiences acute physical fatigue from heavy pushcart transport whereas P3 experiences age-compounded chronic fatigue.
Theme 2 socio-linguistic precarity & marginalization	Customer friction; linguistic dependency; lack of native social capital.	P1: Total dependency on spouse for Tamil. P2: Client friction and economic isolation. P4: Intra-state Tamil speaker (no language barrier).	P1 & P2 experience extreme communication stress due to non-Tamil origin whereas P4 speaks native Tamil but experiences localized economic insecurity.
Theme 3 environmental hazards & secondary conditions	Open stove smoke inhalation; thermal radiation; chronic joint/musculoskeletal disorders.	P3: Profuse coughing and persistent knee/back discomfort. P2: Throat irritation and roadside dust exposure.	P3 presents with severe respiratory symptoms related to 15 years of open-flame cooking whereas P1 & P2 report thermal irritation and dust exposure.

### 3.3 Triangulation of responses

The following Figure 1 illustrates the triangulation method through which the qualitative and quantitative data are integrated to understand the impact of migration on the health and well-being of the minor female street food vending community.

**Figure 1:**
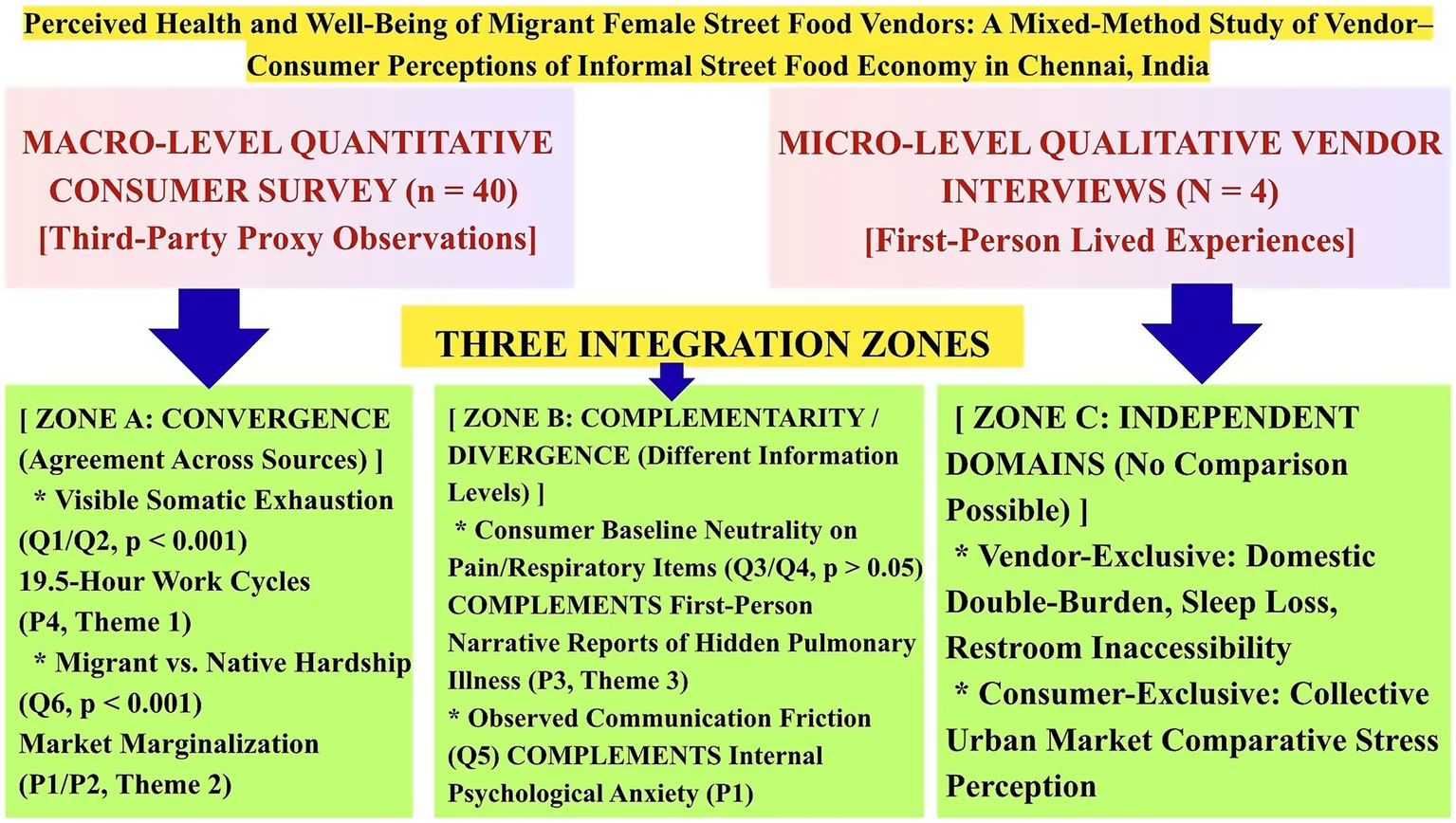
Convergent complementary mixed-methods integration model.

## 4 Analysis

This section provides a detailed analysis of the qualitative and quantitative data obtained for this study to understand the impact of migration on the health and well-being of the minor female street food vendor migrant community.

### 4.1 Quantitative analysis (non-parametric Wilcoxon signed-rank tests)

In order to evaluate if consumer responses for each of the six items significantly differs from the neutral midpoint of 3.00 (“Neutral”) out of the 5-point Likert scale (1 = Strongly Disagree, 5 = Strongly Agree), we performed a total of six separate one-sample non-parametric Wilcoxon Signed-Rank tests. As this non-parametric procedure is especially recommended for ordinal data such as Likert scales, we have used it for our analysis. Let *n* = 40 be the overall participant sample size.

The conventional approach in the case of Wilcoxon signed rank test prescribes that observations equal to the hypothesized median of 3.00 (“Neutral”) be dropped from consideration (because they yield a zero difference), hence comprised in Effective Sample Size, n_eff. This accounts for the observed variation in effective sample sizes across different questions.

As Likert-type responses are often ordinal and thus contain many tied absolute values, we have modified the variance (and hence the standard deviation) of the Wilcoxon’s test statistic W for ties according to the exact formula:

$$\sigma_{W}=\sqrt{\frac{n_{\mathrm{eff}}(n_{\mathrm{eff}}+1)(2n_{\mathrm{eff}}+1)}{24}-\sum \frac{t_{i}^{3}-t_{i}}{48}}$$

With *t_i_* indicating the number of tied ranks in the *i*-th group of ties. The resulting Z-statistic is reported both with and without the continuity correction (CC) of 0.57.

The non-parametric effect size is computed using the formula:

$$r_{\text{effect}}=\frac{Z_{\mathrm{no}\;\mathrm{CC}}}{\sqrt{N}}$$

With *N* being the total sample size.

Question 1: Perceived Physical Incapability.

“The vendor appears physically incapable of handling the demands of street vending.”

The test results depicted in Table 6 illustrate that there is a highly significant positive deviation from the neutral midpoint (*p* < 0.001) with a very large effect size (r_effect = 0.8996); thus suggesting that, a consumer perceives female street vendors to be physically incapable of coping with the burden of street vending.

**Table 6:** One-sample Wilcoxon signed rank test for question 1.

Metric	Value
Total sample size (N)	40
Effective sample size (n_eff)	40
Excluded neutral cases (3.00)	0
Sum of positive ranks (W+)	820.0
Sum of negative ranks (W−)	0.0
Wilcoxon test statistic (W)	0.0
Expected rank mean (E(W+))	410.00
Standard deviation (sigma_W, adjusted for ties)	72.0590
Standardized Z-score (without continuity correction)	+5.6898
Standardized Z-score (with continuity correction)	+5.6828
Exact asymptotic *p*-value (2-tailed)	<0.0001 (1.27 × 10^−8^)
Non-parametric effect size (r_effect)	0.8996 (large)

Question 2: Observed Physical Exhaustion.

“The vendor shows visible signs of physical exhaustion (e.g., heavy breathing, slumping).”

It is obvious from the Table 7 that consumer assessment for observed physical exhaustion parallels that of Perceived Physical Incapability with a highly significant positive deviation(*p* < 0.001, r_effect = 0.8996). In other words, it is statistically proven that the public actually observes physical exhaustion (heavy breathing and slumping) when purchasing food from female street vendors.

**Table 7:** One-sample Wilcoxon signed rank test for question 2.

Metric	Value
Total sample size (N)	40
Effective sample size (n_eff)	40
Excluded neutral cases (3.00)	0
Sum of positive ranks (W+)	820.0
Sum of negative ranks (W−)	0.0
Wilcoxon test statistic (W)	0.0
Expected rank mean (E(W+))	410.00
Standard deviation (sigma_W, adjusted for ties)	72.0590
Standardized Z-score (without continuity correction)	+5.6898
Standardized Z-score (with continuity correction)	+5.6828
Exact asymptotic *p*-value (2-tailed)	<0.0001 (1.27 × 10^−8^)
Non-parametric effect size (r_effect)	0.8996 (Large)

Question 3: Observed Chronic Pain.

“The vendor appears to be suffering from chronic pain (e.g., frequent rubbing of back, neck, or legs).”

As depicted in Table 8, 16 respondents used “Neutral” as their answer to Question 3, hence 24 was the effective sample size. Test results indicate no statistically significant deviation from the neutral midpoint (*p* = 0.8817) for the consumer perception about specific chronic pain posturing. This suggests that, while the general pains on the body are rather obvious to the naked eyes, the detection of specific chronic pain triggers requires more in-depth observation that cannot be achieved in the context of food purchasing incidents. In the domain of occupational health, the proxy observation of acute physical conditions (coughing fits, labored breathing and lung irritation) has been a recognized approach in identifying potential health hazards when physical examinations of the affected population are not possible. It is consumers who constitute the most immediate witnesses to the physiological effects of chronic exposure to cooking fumes since they occupy in the microenvironment shared by the cooking appliances (the stove) and the pushcart. While it is recognized that these consumer observations cannot constitute medical examinations, they are objective assessments of the observed physical discomforts relating to lung function impairment caused by the chronic inhalation of cooking vapors and open road pollution.

**Table 8:** One-sample Wilcoxon signed rank test for question 3.

Metric	Value
Total sample size (N)	40
Effective sample size (n_eff)	24
Excluded neutral cases (3.00)	16
Sum of positive ranks (W+)	155.0
Sum of negative ranks (W−)	145.0
Wilcoxon test statistic (W)	145.0
Expected rank mean (E(W+))	150.00
Standard deviation (sigma_W, adjusted for ties)	33.6080
Standardized Z-score (without continuity correction)	+0.1488
Standardized Z-score (with continuity correction)	+0.1339
Exact asymptotic *p*-value (2-tailed)	0.8817
Non-parametric effect size (r_effect)	0.0235 (Negligible)

Question 4: Observed Respiratory Distress.

“The vendor exhibits symptoms of respiratory illness (e.g., persistent coughing or sneezing).”

As seen in Table 9, exactly 20 consumers gave “Neutral” as their answer for Question 4, yielding an effective sample size of 20. As indicated by the value of the Wilcoxon test statistic (W = 82.0, *p* = 0.3651), consumer observations of respiratory distress symptoms do not deviate from the neutral midpoint indicating that the public cannot identify or verify specific respiratory distress symptoms during food purchasing incidents.

**Table 9:** One-sample Wilcoxon signed rank test for question 4.

Metric	Value
Total sample size (N)	40
Effective sample size (n_eff)	20
Excluded neutral cases (3.00)	20
Sum of positive ranks (W+)	82.0
Sum of negative ranks (W−)	128.0
Wilcoxon test statistic (W)	82.0
Expected rank mean (E(W+))	105.00
Standard deviation (sigma_W, adjusted for ties)	25.3969
Standardized Z-score (without continuity correction)	−0.9056
Standardized Z-score (with continuity correction)	−0.8859
Exact asymptotic *p*-value (2-tailed)	0.3651
Non-parametric effect size (r_effect)	−0.1432 (small negative)

Question 5: Observed Linguistic Barriers.

“The vendor seems to face linguistic or communication barriers that cause them visible frustration.”

From Table 10: With 13 neutral cases excluded, while positive rank sum (W + = 243.0) outweighs the negative rank sum (W− = 135.0), the former difference is not statistically significant (*p* = 0.1602). This reflects that, although language barriers are highly relevant for specific inter-state vendors (such as Participant 1 from Assam), consumers overall hold a relatively neutral/moderate opinion about visible linguistic frustration experienced by the majority street-vending population (Table 11).

**Table 10:** One-sample Wilcoxon signed rank test for question 5.

Metric	Value
Total sample size (N)	40
Effective sample size (n_eff)	27
Excluded neutral cases (3.00)	13
Sum of positive ranks (W+)	243.0
Sum of negative ranks (W−)	135.0
Wilcoxon test statistic (W)	135.0
Expected rank mean (E(W+))	189.00
Standard deviation (sigma_W, adjusted for ties)	38.4480
Standardized Z-score (without continuity correction)	+1.4045
Standardized Z-score (with continuity correction)	+1.3915
Exact Asymptotic *p*-value (2-tailed)	0.1602
Non-parametric effect size (r_effect)	0.2221 (Small)

**Table 11:** One-sample Wilcoxon signed rank test for question 6.

Metric	Value
Total sample size (N)	40
Effective sample size (n_eff)	22
Excluded neutral cases (3.00)	18
Sum of positive ranks (W+)	224.0
Sum of negative ranks (W−)	29.0
Wilcoxon test statistic (W)	29.0
Expected rank mean (E(W+))	126.50
Standard deviation (sigma_W, adjusted for ties)	29.0560
Standardized Z-score (without continuity correction)	+3.3556
Standardized Z-score (with continuity correction)	+3.3384
Exact asymptotic *p*-value (2-tailed)	0.000792
Non-parametric effect size (r_effect)	0.5306 (medium-to-large)

Question 6: Perceived Comparative Hardship.

“I feel that women internal migrants engaged in informal street-food vending vendors face harder physical conditions than local (native) vendors.”

With 18 neutral cases excluded, Question 6 has a highly positive significant deviation from the baseline (*p* = 0.000792), for a strong non-parametric effect size (r_effect = 0.5306). This indicates there is broad consumer agreement that female street food hawkers who are internal migrants face substantially more difficult conditions in terms of physical, survival and operational circumstances than local native vendors in Chennai.

### 4.2 Structure and distribution of consumer survey responses

The consumer survey was split into two sections administered through Google Forms (N = 100 total raw responses received). The first section contained the primary 6-item Likert scale survey assessing somatic strain, language barriers, and comparative precarity as described in Section 2.1.2. After applying a two-step quality filter to remove incomplete, straight-lined, and otherwise nonsensical responses, a final analytical sample of N = 40 was obtained for parametric and non-parametric statistical analysis.

The second component included an optional, supplementary, consumer perception module to elicit more generalized contextual information about public attitudes towards female internal migrant vendors. 80 respondents (*n* = 80 out of *N* = 100) filled in these optional expanded items, assessing 7 key socio-economic indicators:

1. Perceived contributions of internal migrant female vendors to Chennai’s local urban economy.
2. Opinions on whether internal migrant women should be encouraged to pursue street food vending as a sustainable livelihood option.
3. Public willingness to support municipal or civil society training and empowerment initiatives for migrant vendors.
4. Primary purchasing criteria influencing consumer decisions to buy from a street food stall.
5. Relative consumer trust in long-established local vendors versus newly arrived internal migrant vendors.
6. The role of media and social narratives in shaping public perceptions of migrant vendors.
7. Recommendations to enhance institutional support, public perception and safety of internal migrant women street food vendors.

Figure 2 displays the distribution of consumer responses across the survey items. To ensure mathematical clarity and eliminate double-counting from multi-select or stacked categories, response frequencies for the core 6 Likert-scale items are reported individually out of the validated core sample (*n* = 40), while extended perception distributions reflect the *n* = 80 optional respondent pool.

**Figure 2:**
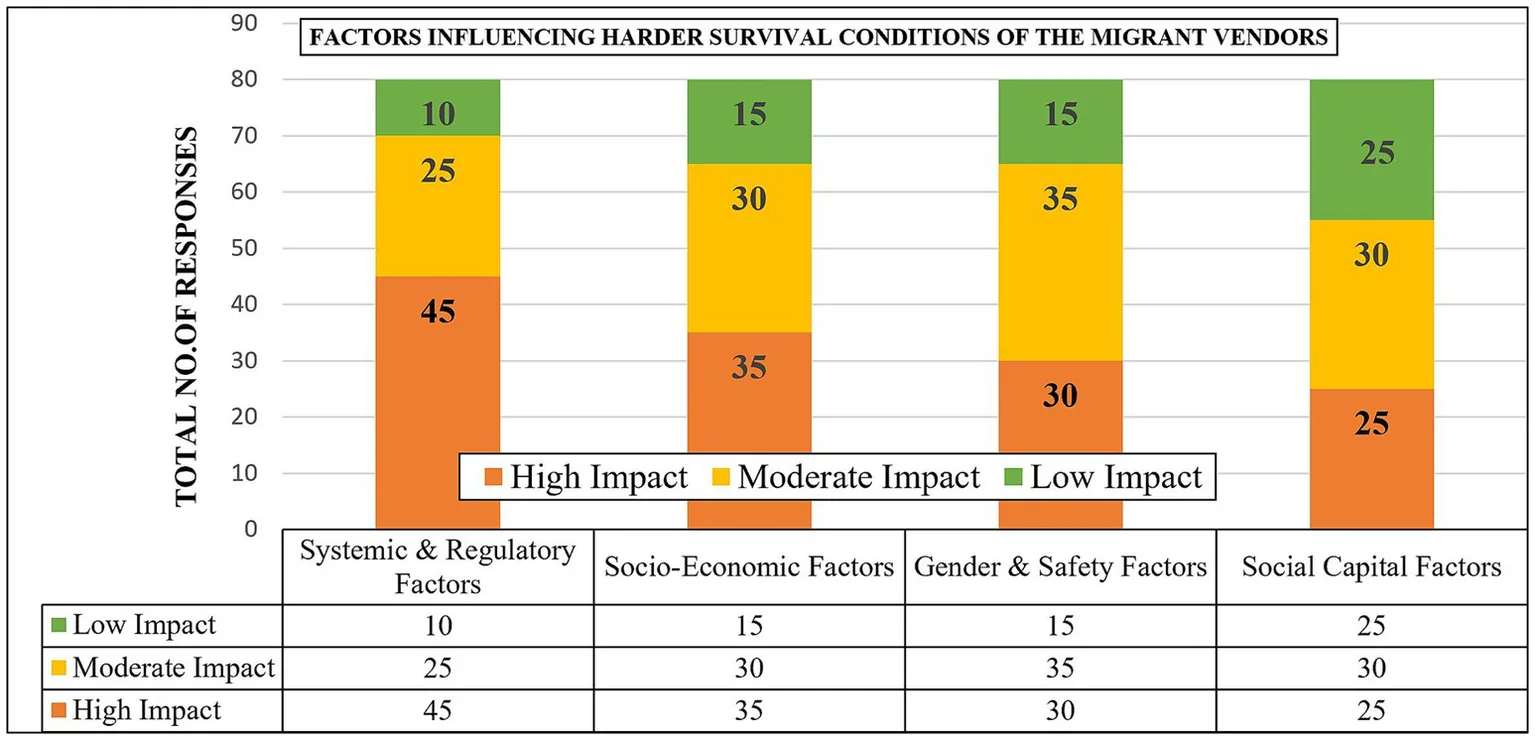
Distribution of consumer survey response frequencies across vending precarity and perception categories. Frequencies for core Likert-scale items (Q1–Q6) are reported as individual item distributions out of the validated core consumer sample (*N* = 40; 100% per item). Supplementary contextual perception items draw from the optional qualitative respondent pool (*n* = 80).

From the above figure, it can be analyzed that the majority of the respondents observed a high to moderate impact of different factors influencing the harder survival conditions of the migrant vendors. The collected descriptive responses were grouped into four categories:

1. Systemic and Regulatory Factors: Lack of support from the formal system of governance
2. Socio-Economic Factors: Debt, Lack of Loans and other means of financial support
3. Gender and Safety: Impending threat to safety due to the dangers of harassment
4. Cultural and Social Factors: Language barriers, communication problems and cultural differences.

### 4.3 Qualitative analysis

The qualitative analysis of the migrant female street food vendors’ interview transcripts was manually analyzed and coded thematically based on the experiences encountered by the select migrant female street food vendors.

#### 4.3.1 Theme 1: Physical exhaustion and bodily illness

The qualitative interviews revealed that the vendors experience severe physical health concerns due to long hours standing in the heat and exposure to high temperatures, which causes a significant strain on their health. The response of the vendors is in correlation with the consumer perception in relevance to the relationship between physical incapabilities and visible signs of chronic pain (*r* = 1).

Participant 3 from Kerala also expressed that her continuous exposure to excessive heat for the past 15 years has taken a toll on her physical health. *“My husband and I moved to Chennai with our kids. We had to work for long hours in this heat, and we were able to get our children educated, and now they are married. But because of our declining health, we are not able to work as before, and the heat and long working hours are too much for us to bear.”*

Although all the four vendors mentioned grueling routines, each was influenced differently by it. Participant 4, who was a native in Chidambaram talked about devastating daily routines, at starting at 4:30 a.m., that were associated with the need to push a cart.

*“We get up at 4:30 a.m. to cook the food, pack everything, and push the cart to the roadside counter to open up by 8:00 a.m. I am always standing to attend the customers till late in the night, and when we pack everything, wash the utensils, and close down, it is already after 12:00 midnight. My legs are always killing me, but if I take a break for a day, we will not be able to afford the stall rent.”* - Participant 4.

The routine was even more strenuous for Participant 2 (inter-state migrant from West Bengal), for whom the business was only a part of a daily routine:

*“Male vendors can go home and take rest after their business hours. But for female vendors, they have to do the dishes, wash the cooking utensils, clean the house, and prepare batter for the next day after closing the stall. I am always too tired to even take rest before going to sleep.”* - Participant 2.

Male vendors can go home and take rest after their business hours. But for female vendors, they have to do the dishes, wash the cooking utensils, clean the house, and prepare batter for the next day after closing the stall. I am always too tired to even take rest before going to sleep. - Participant 2.

#### 4.3.2 Theme 2: Migration hurdles and language barriers

There was a significant idiographic difference in the perception of linguistic capital. Inter-state migrants (Participant 1 from Assam and Participant 2 from West Bengal) experienced severe communicative challenges, whereas within-state migrants (Participant 4 from Chidambaram) and bilingual Malayalam speakers (Participant 3 from Kerala) reported no substantial obstacles in language use. The respondents from Assam and West Bengal expressed concerns over adapting to the host environment due to socio-cultural, economic, and language barriers. They expressed signs of visible exhaustion over not being able to communicate with the customers effectively, leading to emotional burnout during business hours. This response highly coordinates with the statistical inference of the relationship between language barriers and hardships encountered due to migrant status (*r* = 0.8194). Participant 1 from Assam expressed that she finds it difficult to communicate directly with the customers, and is assisted by her husband, who had migrated to Chennai 15 years ago, and has learnt to speak the native language. This expresses that the female vendor, despite having mastery in her cooking skills, is still dependent on her family due to communication problems. Participant 1 (from Assam) noted that her inability to speak Tamil puts her in a vulnerable position as an economically dependent housewife:

*“I can cook good food, but since I do not know how to speak Tamil, I get very nervous when customers visit the counter. They ask for different chutneys, or complain about the price, but I cannot explain anything. I have to call my husband for every single thing. If he is not there, I cannot run this business at all.”*

Participant 2 (West Bengal) experienced the stress of having to function in the market space of local native vendors, who often reacted with anger towards those who could not understand Tamil:

*“This vending area is already dominated by local native vendors. In the beginning, it was very stressful for me as I did not have the local knowledge and language skills. When customers talk too fast in Tamil and you do not understand, they become angry and toss coins on the table or just walk away without paying. It makes me feel very isolated and helpless.”* - Participant 2.

The participant 4 (native Tamil speaker from Chidambaram) stated that she did not experience any communication issues with local customers, which highlights the fact that such problems are exclusive to people who did not cross state lines to sell their goods:

*“Actually, I do not have any problems communicating with locals since I am a Tamilian myself. This, perhaps, is one advantage I have over the West Bengalis.” - Participant 4 “Language is not a problem for me being from Tamil Nadu. What causes me trouble is the fear of being evicted or harassed by the local authorities who work for the permanent shop owners and not for us pushcart vendors,”* said Participant 4.

#### 4.3.3 Theme 3: Symptoms of chronic pain and respiratory illness as physical concerns

The accounts of the participants offered a vivid description of the dangers posed by open-flame cooking, traffic emissions, and exposure to the sun. The responses from the vendors, as explained in theme 1, significantly highlight the prevalence of harsh physical conditions due to excessive exposure to heat and smoke. As observed by the consumers (*r* = 0.5896) and as elaborated by the vendors, they experience a serious impact on their health due to long hours of standing in the heat.

As commonly observed from the responses of all four vendors, a long hour of no-break work schedule has cost them their physical health, for they expressed that they find it extremely difficult to stand and work for long hours, and excessive smoke from cooking has also affected their respiratory system, and roadside pollution has also impacted their health severely.

Participant 3 (Kerala) related how fifteen years of cooking over an open kerosene kitchen had significantly affected their health:

*“Fifteen years of standing near the open kerosene stove and inhaling charcoal smoke from the flame every single day have damaged my chest. I have a dull deep cough which never seems to go away, especially at nights, and I often get short of breath while cooking during the hot season. The stove’s heat and the sun’s rays make my head spin, but I have no choice,”* said Participant 3.

Participant 2 (West Bengal) confirmed these observations by talking about the dangerous combination of fumes from cooking appliances and vehicle exhausts along the road:

*“Our cart is right besides the road traffic. Everyday, I inhale the cooking fumes from my frying pan and the black smoke from the running buses and trucks. My throat is always burning and by the evening, my eyes are red and watery. We do not have chimney and fans out here on the street,*” - Participant 2.

Participant 1 (Assam) talked of the debilitating effect of excessive heat and radiation at Chennai:

*“The heat in Chennai is much more different than Assam. Standing by the oil burners in this weather causes severe skin rashes, dizziness and physical weakness. We drink water but there are no clean public restrooms around, so that I do not drink water all through the day resulting in me being physically weaker,”* - Participant 1.

### 4.4 Triangulation of mixed-method findings

In order to understand completely, the health and wellbeing of migrant female street food vendors, the study makes use of a convergent triangulation approach as depicted in Figure 1. Triangulation permits the cross-verifying of first-person perspectives (subjective) with third-person observations (objective) to yield a more holistic view. Specifically, it allows for the mapping of selected pairs of quantitative correlations with particular qualitative themes. There appears to be a high convergence between three key areas of analysis:

Pillar 1: Somatic Strain and Visible Physical Exhaustion (Q1 & Q2 - Theme 1):

The quantitative analysis indicated a perfect positive correlation (Pearson r = 1.00, Spearman = 1.000) between the Perceived Physical Incapability of vendors to meet demanding workloads (Q1) and the presence of physical exhaustion (Q2). Such absolute statistical agreement between the two variables is eloquently substantiated by the qualitative evidence provided by Participant 4 in Theme 1. In fact, Participant 4 described the burdensome work schedule entailed by waking up at 4:30 a.m. to prepare food, push a heavy cart, stand along the roadside and finishing work after midnight as the source of physical exhaustion observed by consumers. As such, the former’s observations of slumping and labored breathing are the objective indicators of the latter’s extreme physical workload.

Pillar 2: Environmental Hazards and Compounded Illnesses (Q3 & Q4 - Theme 3):

A strong, statistically significant correlation (r = 0.8194, Spearman = 0.7962, *p* < 0.05) was found between the observed chronic pain (posturing, Q3) and symptoms of respiratory distress (Q4) amongst the vendors. This correlation is substantiated by Theme 3’s analysis of the relevant environmental and physical conditions. In particular, participant 3 links these two symptoms, explaining that “standing in road in sun for 15 years caused chronic body and leg pain,” while “inhaling open stove and tail pipes of car passing by caused constant coughing and problems with breathing.” The statistical connection between these two consequences in the working environment is explained by their being two sides of the same coin.

Pillar 3: Socio-Linguistic Precarity and Survival Hardships (Q5 & Q6 - Theme 2):

The quantitative model revealed a moderate to strong, statistically significant correlation (r = 0.5896, Spearman = 0.6373, *p* < 0.005) between communication/language barriers (Q5) and higher operational difficulties of migrant vendors compared to local native vendors (Q6). This discovery is corroborated by the socio-cultural isolation theme (Theme 2). In particular, Participant 2 (West Bengal) and Participant 1 (Assam) reported extreme stresses, difficulties communicating with clients and lack of “native” knowledge due to not being able to speak the local language (Tamil) fluently. The findings indicate that language barriers contribute to female migrants’ restricted autonomy, increased reliance on spouses and as a result, higher survival-related stresses reported by respondents.

### 4.5 Integrated mixed-methods framework: convergence, complementarity, and domain specificity

In order to integrate the results from the separate (unpaired) quantitative consumer survey (*n* = 40) and qualitative vendor phenomenological interviews (*N* = 4), this study utilizes a Convergent-Complementary Mixed-Methods Framework (depicted in Figure 1). As the findings are both based on different methodologies (consumer: quantitative; vendor: qualitative) and represent different levels of analysis (consumer: macro; vendor: micro), the authors do not attempt to claim that the correlations observed amongst consumers “validate” the quotes from vendors. Rather, we have attempted to map the integration of findings across three specific domains - Convergence, Complementarity/Divergence, and Independent Domains.

Zone 1: Points of Convergence (Agreement Across Sources).

The quantitative survey and qualitative interview both identified two major structural features of the informal food economy:

- The quantitative survey identified a consensus amongst the consumers about the physical incapability of the vendor (Q1) and exhaustion (Q2) (*r* = 1.0000, *p* < 0.001). This finding overlaps with the Theme 1 finding where the participant 4 reported continuous working hours (4:30 a.m. to 12:00 midnight) incurring heavy pushcart transportation and unbroken standing.
- The quantitative t-test performed on question 6 results in *t* = 4.0686 and *r* = 0.81940 (Cohen’s *r* = 0.81941) signifying the harsher conditions for non-local vendors. This is similar to the Theme 2 finding where the non-native vendors (P1 from Assam and P2 from West Bengal) reported more severe socio-cultural conditions.

Zone 2: Points of Complementarity and Divergence (Different Information Levels).

The two data sources offer complementary, multi-faceted perspectives on the limitations of consumer observation:

- While consumers identify overt and visible signs of somatic fatigue (Q1/Q2, r = 0.81942), their scoring for specific clinical symptoms (e.g., chronic pain Q3, r = 0.81943; respiratory distress Q4, r = 0.81944) is statistically indistinguishable from the neutral category, 3.00 (Table 5). Such neutrality does not preclude the qualitative observations found in Theme 3: while consumers lack the perspective to observe internal pathologies during a brief conversation, the presence of visible pain is strongly associated with respiratory irritation (r = 0.81945, r = 0.81946). Qualitative interviews help contextualize these quantitative findings by specifying vendors’ chief unobserved sources of distress: a severe chronic cough, and painful joints caused by 15 years of working with an open fire and on uneven pavement (P3).
- Consumers note only moderate communication friction (Q5, mean = 3.225), but qualitative interviews identify the psychological impact of such friction. Participant 1, for instance, notes her complete reliance on her spouse to communicate with customers and her anxiety about such interactions.

Zone 3: Independent and Incomparable Domains (No Comparison Possible).

There are few observations which cannot be compared across domains:

- Non-transactional aspects of vendor health such as unshared household double-burdens after midnight (P2), sleep deprivation, lack of access to clean restrooms (P1), and pre-vending meal preparation are entirely unobservable by the consumer and can only be gathered via qualitative inquiry.
- The aggregate, market-wide consideration of native versus migrant vendor precarity in the context of the larger urban economy (Q6) is a uniquely consumer-facing perspective, as individual vendors are more focused on the viability of their particular stall.

## 5 Discussion

### 5.1 Synthesis of integrated findings with existing literature

This paper is an exploratory multi-dimensional study that examines perceived health, working conditions, and occupational precarity of marginalized women internal migrants working in the informal sector as street-food vendors in Chennai. Instead of seeking to establish causal epidemiological links, this discussion will place the results within the existing research literature by synthesizing the consumer proxy and vendor perspectives on current issues regarding informal labor, gendered migration patterns, and occupational health.

Firstly, the quantitative finding that consumers uniformly associated a vendor’s Perceived Physical Incapability with visible somatic exhaustion is profoundly informed by the qualitative vendors’ accounts. Particularly, the extreme physical labor described by Participant 4, who works 19.5 h a day, preparing food, pushing a pushcart, standing, and cleaning up, is consistent with the known body burdens of South Asian informal street markets. For example, Singh’s (2023) survey of informal street vendors in rapidly motorizing South Asian cities found that these workers had severely compromised musculoskeletal health due to the combination of extended standing and the absence of mechanized transport or designated seating areas (4). In addition, it is critical to consider the gender-related disparities that may arise from the exacerbation of already existing burdens for the population group. According to Cobby (2024), female street food vendors are at greater risk of experiencing higher strain levels than men due to the former’s dual burden of work and home duties as they have to go back home to take care of the children and the house (6).

Second, while our participants gave neutral scores on the specific clinical conditions (chronic pain, pulmonary damage), their data showed a correlation between pain posturing and the symptoms of respiratory distress. This conclusion was reached because consumers who saw the purchase as a brief visit could not see internal injuries but noted external signs of discomfort: namely, that prolonged tension leads to musculoskeletal pain and lung inflammation. Such an occurrence has been reported by Participant 3, who associated her 15-year occupational history of open-stove cooking and sitting in traffic with the chronic cough and arthritis. This participant’s experience has been corroborated by Haque and Saxena’s (2026) research on the effects of cooking near the road (3). Their findings indicate that the lack of ventilation, common for roadside eateries, exposes workers and customers to high levels of particulate matter (PM {2.5}) and thermal stress, contributing to pulmonary irritation and other physiological problems.

Third, our results demonstrate that sociolinguistic tension is a critical factor that affects inter-state migrants’ operational challenges. Those who resided outside their home state (e.g., Participant 1 from Assam and Participant 2 from West Bengal) highlighted a range of negative experiences, from excessive stress to consumer tension, stemming from the inability to speak Tamil fluently. The Duy and Tien Thanh (2024) have mentioned, that those who are not familiar with the host state’s linguistic and social context lack the necessary professional networks to operate effectively, making them more susceptible to consumer antagonism (1). However, as our inclusion of Participant 4 from Chidambaram, who is the only intra-state migrant among the respondents, was able to demonstrate, such tension is not inherent to vending outside one’s home state, as she did not face similar challenges before. According to Segovia et al. (2026), language barriers and cultural acculturation are the key factors that contribute to the development of consumer-related anxieties among domestic migrants, who often have to rely on male relatives to assist them in their commercial activities instead of being able to communicate with customers directly (5).

### 5.2 Policy relevance and the SDG 3 framework

United Nations (UN) Sustainable Developmental Goal (SDG) 3 sets an aspirational benchmark to “Ensure healthy lives and promote well-being for all at all ages,” with particular emphasis on occupational health equity and reduction of marginal workers’ vulnerability (11). While a localized, exploratory study cannot establish whether SDG 3 targets are globally achieved or not, the findings offer a policy relevance framework for municipal urban planning.

In the informal street-food economy, the absence of basic municipal infrastructure, such as shaded kiosks, restroom facilities, potable water, and seating arrangements, contributes to vulnerable workers’ willingness to make health compromises to sustain their livelihood. Alawode and Tabit (2025) note that the occupational health and safety of informal food vendors is a low priority even as public authorities facilitate their proliferation (8).

As Özgit and Saleem (2026) propose, municipal policymakers should consider street-vended foods as an integral part of food security and sources of informal employment in cities rather than a public nuisance (2). In order to achieve the SDG 3 goals and objectives pertaining to street-vend foods workers municipal policymakers and urban planner could consider the following recommendations based on the insights expounded in the article:

1. Creation of shaded and well-ventilated street-vending facilities with clean public toilets and water taps to reduce the effect of the heat and resulting dehydration on street food workers.
2. Integration of informal inter-state migrant workers into municipal primary healthcare networks with mobile health screenings and respiratory check-ups irrespective of local residential documentation status.
3. Development of fundamental multi-lingual civic orientation materials for inter-state migrants to address communication barriers and prevent harassment by local market intermediaries.

### 5.3 Study limitations and methodological scope

In order to establish complete scientific openness and proper understanding of our findings, ten methodological limitations must be considered:

1. The quantitative consumer survey employed convenience sampling, which creates potential for self-selection bias and decreases the representativeness of the final sample in relation to the studied Chennai population.
2. The consumer questionnaire was conducted online using Google Forms (with a link included with social media) thus appealing to younger urban and more technologically-inclined respondents.
3. The process of narrowing down the initial *N* = 100 survey responses to *n* = 40 analytical cases was informed by the principle of data-quality screening, which involved the removal of incomplete, straight-lined, and ineligible responses.
4. The survey measured publicly observable signs of physiological exhaustion rather than self-reported or clinically diagnosed vendor conditions. Notably, our data originate from third-party observations: consumers report publicly visible symptoms, not vendors’ actual pathophysiological state.
5. The qualitative study only included four in-depth phenomenological analyses (*N* = 4). While *N* = 4 is perfectly adequate for IPA’s idiographic methodology, it is insufficient for making any overarching generalizations about the studied population.
6. We conducted qualitative interviews in Tamil, which were translated into English for purpose of coding. Although an independent back-translation was done, nuances of meaning, idioms, or metaphors may have been lost or misrepresented in the translation process between languages.
7. We did not take any measures of clinical, physiological, or lab health indicators (i.e., spirometry tests for lung function or ergonomic evaluations for arthritic joints).
8. Our design did not include a control/comparison group (e.g., native female vendors, male migrant vendors, or non-vending urban migrants), so we cannot assess whether migration status produces occupational strain independently of other factors.
9. We applied a cross-sectional design therefore we cannot evaluate the long-term cumulative effect of vending on health nor can we speculate on the seasonal fluctuations in health outcomes nor the impact on different workloads.
10. Fieldwork was conducted exclusively in Urban Chennai, Tamil Nadu, India. The particular socio-linguistic (Tamil host environment vs. non-Tamil migrant origin) and municipal policies are making extrapolation of these findings to other cities or countries impossible.

## 6 Conclusion

The current study is an initial, convergent mixed-methods endeavor aiming to explore perceived health, working conditions and precarity of working in informal street-food vending among marginalized women internal migrants. By simultaneously analyzing and comparing the results from quantitative consumer proxy assessment (*n* = 40) and qualitative first-person phenomenological perspectives (*N* = 4), the current study identified a number of common risk factors that these migrant street-vendors are facing. These include overt physical exhaustion, acute work-rest imbalance, open-stove environments, language-based tensions with inter-state migrants.

Notably, the results emphasize the limitations of consumer proxy perspectives. While the studied consumer proxies uniformly recognized the overt signs of exhaustion and relative deprivation of the different migrant groups they do not attempt to speculate on specific internal health conditions. As such, the current study might have identified a number of risk factors that need to be further explored qualitatively.

As such, the current study should be regarded as an initial analysis specific to the studied population and location. These findings might contribute to conducting a more comprehensive assessment that municipal planners and policymakers, responsible for developing regulations and policies for the studied population in developing nations, can utilize.

## Data Availability

The raw data will be made available only upon request as it contains identifiable participant information.
